# Predictive Monitoring of Critical Cardiorespiratory Alarms in Neonates Under Intensive Care

**DOI:** 10.1109/JTEHM.2019.2953520

**Published:** 2019-11-22

**Authors:** Rohan Joshi, Zheng Peng, Xi Long, Loe Feijs, Peter Andriessen, Carola Van Pul

**Affiliations:** 1 Department of Electrical EngineeringEindhoven University of Technology 5612 AZ Eindhoven The Netherlands; 2 Department of Family Care SolutionsPhilips Research 60994 5656 AZ Eindhoven The Netherlands; 3 Department of Industrial DesignEindhoven University of Technology 5612 AZ Eindhoven The Netherlands; 4 Department of NeonatologyMáxima Medical Center 5504 DB Veldhoven The Netherlands; 5 Department of Clinical PhysicsMáxima Medical Center 5504 DB Veldhoven The Netherlands; 6 Department of Applied PhysicsEindhoven University of Technology 5612 AZ Eindhoven The Netherlands

**Keywords:** Alarm fatigue, medical devices, machine learning, NICU, patient monitoring, predictive monitoring, real-time monitoring

## Abstract

We aimed at reducing alarm fatigue in neonatal intensive care units by developing a model using machine learning for the early prediction of critical cardiorespiratory alarms. During this study in over 34,000 patient monitoring hours in 55 infants 278,000 advisory (yellow) and 70,000 critical (red) alarms occurred. Vital signs including the heart rate, breathing rate, and oxygen saturation were obtained at a sampling frequency of 1 Hz while heart rate variability was calculated by processing the ECG – both were used for feature development and for predicting alarms. Yellow alarms that were followed by at least one red alarm within a short post-alarm window constituted the case-cohort while the remaining yellow alarms constituted the control cohort. For analysis, the case and control cohorts, stratified by proportion, were split into training (80%) and test sets (20%). Classifiers based on decision trees were used to predict, at the moment the yellow alarm occurred, whether a red alarm(s) would shortly follow. The best performing classifier used data from the 2-min window before the occurrence of the yellow alarm and could predict 26% of the red alarms in advance (18.4s, median), at the expense of 7% additional red alarms. These results indicate that based on predictive monitoring of critical alarms, nurses can be provided a longer window of opportunity for preemptive clinical action. Further, such as algorithm can be safely implemented as alarms that are not algorithmically predicted can still be generated upon the usual breach of the threshold, as in current clinical practice.

## Graphical abstract

We present a method for reducing alarm fatigue in neonatal intensive care units using machine learning for the early prediction of critical cardiorespiratory alarms. During this study in over 34,000 patient vital sign monitoring hours in 55 infants, 278,000 advisory (yellow) and 70,000 critical (red) alarms occurred. The results indicate that based on predictive monitoring of critical alarms, nurses can be provided a longer window of opportunity for preemptive clinical action, while alarms that are not algorithmically predicted can still be generated as in usual clinical practice.

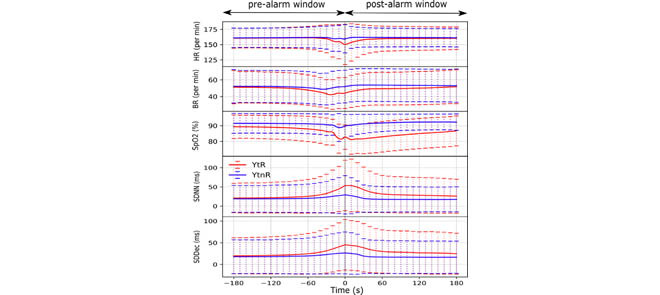

## I. Introduction

Continuous cardiorespiratory monitoring of neonates under intensive care includes electrocardiography (ECG), impedance pneumography and pulse oximetry to estimate the heart rate (HR), breathing rate (BR) and oxygen saturation (SpO2) respectively. Whenever these vital signs breach predetermined thresholds, alarms are generated to redirect the attention of caregivers to the clinical status of the infants. Typically, based on urgency, there are 2 types of alarms – advisory and critical. Advisory alarms, also known as yellow alarms, are generated when vital signs breach a predefined threshold and enter into a physiologically undesirable range while critical or red alarms, generated upon the breach of a second threshold, reflect a potentially dangerous physiological state. Since the consequences of a missed alarm can be severe, patient monitoring errs on the side of caution with alarms typically being generated immediately upon the breach of preset thresholds or after a short delay, for instance, 10s, to allow physiological parameters to auto-correct.

Excessive alarms, a large number of which may be clinically irrelevant, leads to desensitization, and the phenomenon of alarm fatigue wherein clinicians have a delayed or even no-response to alarms [1]–[4]. The situation of alarm fatigue has devolved to a state where alarms, although responsible for safeguarding the health of patients, are also one of the top patient-safety hazards in hospitals [5], [6]. This situation is not surprising since alarm pressure can be massive and a response to each alarm unrealistic within the constraints of the typical clinical workflow. For instance, clinical audits in different neonatal intensive care units (NICU) have found that the daily alarm pressure due to patient monitors ranges between 180-320 alarms per patient per day with yellow alarms outnumbering red alarms in the ratio of 4:1 to 12:1 [7], [8]. Considering that a nurse is responsible for 2 or more infants, such a nurse would experience an alarm every few minutes. In this scenario, it is not surprising that nurses employ heuristics to determine whether alarms mandate a response. To illustrate this, in a study of nearly 6000 critical (red) alarms, nurses responded to only about a quarter of the alarms within 1 minute [4]. This situation is far from ideal since, in certain cohorts of preterm infants, an increased incidence of even short desaturations such as 20s was associated with higher mortality [9]. Furthermore, physiological deterioration in infants characteristically manifests itself in temporally clustered red alarms, often with the simultaneous derangement of multiple vital signs [3]. Such clustering of alarms points to a more severe compromise in physiology wherein a quicker nursing response to the first red alarm may stem the occurrence of subsequent alarms and thereby of large alarm clusters.

In a review by Schmid et al, an overview of approaches based on statistical models and artificial intelligence for the reduction of false alarms is provided [10]. The review highlights that despite the development of such models, these have not been adopted into patient monitoring because of safety concerns and a ‘better safe than sorry’ mentality. In an alternative line of thought, we propose an approach to preempt critical alarms. We hypothesize that by predicting red alarms before they are generated by the current patient monitoring systems, nurses can be provided with a longer window of opportunity for preemptive clinical action. To address this issue, in this paper, we developed a machine learning model for discerning whether a yellow alarm would be followed by a red alarm within a short window of time, for instance, 1 min. Unlike existing alarm systems which are merely threshold-based, our approach is multiparametric and uses data from the window leading up to a yellow alarm. This window of data can be expected to carry more information than a simple breach of the threshold, as has also been suggested elsewhere [11]–[14]. In this predictive alarming approach, it is important to prioritize the specificity of prediction over sensitivity so that the model is deemed reliable by clinicians. For instance, it is preferable to correctly predict only a fraction of yellow alarms that would turn to red (low sensitivity) rather than generate multiple false positives wherein the model predicts red alarms that would never occur (low specificity). Such a modeling approach, based on preempting critical alarms upon the occurrence of a sub-critical physiological deterioration is different from other applications of machine learning for alarm management, the majority of which are based suppressing false alarms [15]–[17].

About yellow alarms, since typically these do not elicit a response, they can be made non-auditory. Doing so though might rob vigilant nurses from being forewarned of an upcoming red alarm. To partially mitigate this concern, we propose an alarm management approach for reducing overall alarm pressure by making all yellow alarms non-auditory in combination with predictive monitoring of red alarms, a proportion of which are generated preemptively at the expense of a small increase in the number of red alarms. Most importantly, the proposed model for alarm prediction would inherently be at least as safe as the current system since red alarms that might be missed by the proposed system would still be generated upon the usual breach of the threshold. It should be noted that in this framework, a yellow alarm merely serves as a subcritical physiological threshold, a breach of which triggers the model to make a prediction of whether a red alarm would soon follow. Instead, an alternative physiological threshold could also be adopted.

## II. Methods and Procedures

### A. Subjects and Setting

In the period between July 2016 and Jan 2018, several clinical studies were conducted in our level III NICU of Máxima Medical Centre, Veldhoven, the Netherlands [16]–[18]. Our NICU has a single patient room design, requiring an extensive monitoring and alarming system (see section B). From these clinical studies, 55 preterm infants were included for predictive modeling. The inclusion criteria were very preterm infants born below 32 weeks of gestation and an expected length of stay of longer than a week. In contrast to the previous clinical studies which included analysis of the data only around skin-to-skin contact between infant and parent (Kangaroo care), the present study used data for the entire period of hospitalization, from admission until discharge.

The characteristics of the study group are provided in Table 1 and are representative of our NICU population. All infants received non-invasive respiratory support (nasal continuous positive airway pressure; high flow nasal cannula) during their NICU stay while for part of their stay, 27% of the infants also received invasive mechanical ventilation. 45% of the infants received surfactant therapy to treat respiratory distress syndrome, and all infants received supplemental oxygen during, at least, part of their stay. Notably, no surgical patients were included as our NICU does not accept surgical cases. The neonatal morbidity was representative of the burden of disease in our NICU population. In 30% of the infants, ibuprofen was used for closure of a patent ductus arteriosus while one-third of all infants received antibiotic therapy for sepsis. Further, 3% of the infants were diagnosed with necrotizing enterocolitis, and approximately 25% of preterm infants were diagnosed with bronchopulmonary dysplasia for which the infants needed supplemental oxygen for at least 28 days. No infant presented with a serious case of a cerebral hemorrhage, i.e., intraventricular hemorrhage grade III or worse. As all data corresponded to routine patient monitoring and was anonymized for retrospective analysis and quality control, no waiver was needed. All parents provided written informed consent analysis.

**TABLE 1:** Characteristics of the Study Population (n = 55). The Postmenstrual Age and the Postnatal Age Were Calculated Based on the Timestamps of Alarms

Characteristics	Median	25th percentile	75th percentile
Birth weight (gram)	955.0	830.0	1085.0
Gestational age (weeks)	27.0	25.86	28.14
Postnatal age (days)	24.0	15.0	37.0
Post menstrual age (weeks)	31.0	29.43	32.57
Duration of monitoring (hours)	433.0	321.0	843.0

### B. Patient Monitoring Data

Non-invasive monitoring of vital cardiorespiratory signs included the HR, BR, and SpO2, measured based on the ECG (3-lead ECG), impedance pneumography and pulse oximetry respectively. These were retrospectively acquired from the patient monitor (Philips IntelliVue MX 800, Germany) at an approximate resolution of 1 Hz via a data warehouse (DWH, IIC iX, Data Warehouse Connect, Philips Medical System, Andover, MA). The raw ECG waveforms (250 Hz) were also extracted and were used to analyze heart rate variability (HRV). Alarms were generated if predefined thresholds were breached – see Appendix for the specific settings. In a single patient room NICU, a distributed alarming system is required to ensure that relevant alarms are transmitted to caregivers who might not be in the room. Specifically, in our NICU, patient monitor alarms, both yellow and red, are generated at the bedside monitor and at the central post. Further, all red alarms are transmitted to the responsible nurses using a wireless handheld device [18].

Logs for all yellow and red alarms were extracted. We defined the *category* of an alarm as the label associated with it in the corresponding alarm log. For instance, desaturation and bradycardia are *categories* of red alarms. Similarly, SpO2-low and SpO2-high are yellow alarm *categories*. All alarms were generated based on monitor settings. The physiological thresholds for generating alarms, including the delay settings and the averaging times are summarized in Appendix.

Typically, red alarms of the category desaturation and bradycardia are preceded by the corresponding yellow alarms, SpO2-low, and HR-low respectively. Since multi-parametric deterioration is common in neonates, it is also possible, for instance, for desaturation to be preceded by both SpO2-low and HR-low alarms.

Certain categories of red alarms such as the apnea alarms do not have a corresponding yellow alarm but may nevertheless be preceded by a yellow alarm such as the SpO2-low. Similarly, the yellow alarm category of SpO2-high does not have a corresponding red alarm. Further, it should be noted that current patient monitors that utilize chest-impedance for monitoring respiration are poor in their ability to detect apneas [19]. Consequently, clinical staff is alerted to apneic episodes due to apnea-associated bradycardia and desaturation events.

### C. Data Selection for Predictive Monitoring of Alarms

Since the goal of this work was to develop a model that would predict red alarms upon the generation of yellow alarms (or alternative predefined thresholds), a natural question arises about what constitutes a meaningful epoch of time preceding the yellow alarm, henceforth called the pre-alarm window, which holds relevant information for prediction. Similarly, what constitutes a suitable post-alarm window in which one would like to predict a red alarm? Based on clinical insights, we empirically chose both the pre- and post-alarm windows to range between 1-3 minutes in length. First, classifiers were developed with the pre-alarm window held constant at 3 minutes while the post-alarm window was changed to 3, 2 and 1 minute. Then, the pre-alarm window was changed to 2 and 1 minute respectively while the post-alarm window was held constant at 1 minute, leading to 5 different classifiers based on different combinations of pre- and post-alarm windows.

All yellow alarms that led to *at least* 1 red alarm, irrespective of the category of the red alarm, within the post-alarm window were termed Yellow-to-Red (*YtR*) alarms and constituted the case-cohort while those yellow alarms that did not lead to a red alarm (*YtnR*) constituted the control cohort and were considered eligible for analysis. To characterize the typical transition times from yellow to red alarms, the cumulative density function of the time to transition from *all* yellow to red alarms was generated, censored at 3 minutes. It should be noted that in the case of *YtR* alarms, more than 1 red alarm might be present in the post-alarm window.

The number of case (*YtR*) and control data (*YtnR*) was dependent on the length of the pre- and post-alarm windows. Due to the technical limitations of data storage and data extraction, ECG data were not available on all days. Alarm data with missing ECGs were discarded. Additionally, those alarm data were also discarded where 30% (or if 10% of consecutive samples) of HR, BR or SpO2 samples were absent in the pre-alarm window. Data coverage for the post-alarm window was 100%. Table 2 characterizes the total number of yellow and red alarms that were generated per infant as well as the number of alarms that were eligible for analysis (valid alarms) corresponding to a pre- and post-alarm window of 3 minutes. The total number of valid alarms, acquired from all infants, for different lengths of pre- and post-alarm windows are also provided in Table 2.

**TABLE 2:** The Number of Yellow and Red Alarms Generated per Infant, Including the Number of Valid Alarms for a Pre- and Post-Alarm Window of 3 Minutes, as Well as the Total Number of Valid Alarms Acquired From all Infants for Different Lengths of Pre- and Post-Alarm Windows

No. of alarms	Median	25th percentile	75th percentile
per infant			
Yellow	1784.0	452.5	6437.5
Red	461.0	210.5	1615.0
YtR	288.0	98.0	1643.0
YtnR	1414.0	352.0	4969.0
YtR (valid^*^)	177.0	80.0	1095.0
YtnR (valid^*^)	878.5	282.25	2716.0
Total number of valid alarms for different lengths of pre- and post-alarm windows			
Pre-alarm window (min)	Post-alarm window (min)	No. ofYtRalarms (valid^*^)	No. ofYtnRalarms (valid^*^)
3	3	47,255	149,007
3	2	40,869	155,387
3	1	31,901	164,352
2	1	31,993	163,637
1	1	32,034	163,065

^*^ Approximately, 70% of the original 278,000 yellow alarms satisfied the data sufficiency criteria in the pre-alarm window and were eligible for inclusion in the analysis. See Methods, section C for a description of the data sufficiency criterion.

### D. Data Preprocessing and Visualization

Since the HR, BR and SpO2 data were acquired from a data warehouse at an approximate resolution of 1 Hz, these parameters were resampled at precisely 1 Hz in the pre- and post-alarm windows using the method of cubic spline interpolation. A peak detection algorithm was used to identify the location of R-peaks in the ECG, following which the interbeat intervals were calculated, similar to previous work [20], [21]. All interbeat intervals longer than 1.5s were removed as potential artifacts before calculating HRV features. All data were analyzed using Python.

To illustrate the underlying data around the alarm, the mean values of the HR, BR, and SpO2 parameters as well as 2 features of HRV, the SDNN and the SDDec (see Table 3 for definitions), are shown for pre- and post-alarm windows of 3 minutes, for both *YtR* and *YtnR* alarms in Fig. 2. The differences between these time-series served as a step toward feature generation.

**TABLE 3:** Description of the Features Used for Developing the Decision Tree Classifiers

Feature category (no. of features)	Feature name abbreviation	Feature description
Infant metadata (3)	GA	Gestational age in days
	BW	Birthweight in gram
	PNA	Postnatal age in days
Yellow alarm category (1)	Y_Alarm_Cat^b^	The yellow alarm category could be one of the following – HR-low, HR-high, SpO2-low, SpO2-high, BP-low, and BP-high.
Alarm counts (2)	Count_Y_Alarm	No. of yellow alarms that occurred in the pre-alarm window
	Count_R_Alarm	No. of red alarms that occurred in the pre-alarm window
HR, BR and SpO2 based features ($3\times 13=39$), with HR, BR, and SpO2 described by Parameter	Parameter_Occ	Feature value at the moment of occurrence of the yellow alarm
	Parameter_Min	Minimum value
	Parameter_Mean	Mean value
	Parameter_Std	Standard deviation
	Parameter_NTC_Y	No. of times the parameter crossed the yellow alarm threshold.^c^
	Parameter_NTC_R	No. of times the parameter crossed the red alarm threshold.
	Parameter_TUR	Cumulative time under red alarm threshold
	Parameter_DI	Delta index – the average of the absolute differences between the mean values of 2 successive and non-overlapping 12s intervals. [31]
	Parameter_CTM	Central tendency measure – the sum of distances to the origin of a second order difference plot of all points except the furthest 5% of all points.
	Parameter_ApEn	Approximate entropy – calculated using a run-length of 2 with a tolerance of 25% of the standard deviation of the data. [32]
	Parameter_LZC	Lempel-Ziv complexity – the median value was used as a threshold for binarization. [33]
	Parameter_Slope^d^	The slope of the regression line fitting the last 50s of data preceding the yellow alarm.
	Parameter_Rvaluee	Coefficient of correlation between actual values and those predicted by regression.
Correlation features of HR, BR and SpO2 ($3\times 2$)	Max_Corr	The maximum cross-correlation between a parameter of window length one-third the length of the pre-alarm window immediately preceding the yellow alarm with the entire pre-alarm window of the other parameter at 5s intervals, without padding. Prior to cross-correlation, the parameters were normalized using the standard score.
	Lag_At_Max_Corr	The lag corresponding to Max_Corr
HRV based features^e^	NN_Occ^f^	NN-interval at occurring moment
	NN_AUC^g^	Area under the NN-intervals.
	SDNN_Occ	Standard deviation of NN-intervals at occurring moment.
	SDNN_AUC	Area under the SDNN time-series.
	RMSSD_Occ	Root mean square of the standard deviation of NN-intervals at occurring moment.
	RMSSD_AUC	Area under the RMSSD time-series.
	pNN50_Occ	Percentage of NN-intervals longer than 50ms at occurring moment.
	pNN50_AUC	Area under the pNN50 time-series.
	pDec_Occ	Percentage of NN-intervals longer than mean value of NN intervals[21]
	pDec_AUC	Area under the pDec time-series.
	SDDec_Occ	Standard deviation of all NN-intervals contributing to pDec[21]
	SDDec_AUC	Area under the SDDec time-series.

^a^All features were calculated using the entire pre-alarm window unless explicitly stated otherwise.

^b^ HR-low, HR-high, SpO2-low, SpO2-high, BP-low, and BP-high constituted all the yellow alarm categories.

^c^ The thresholds for HR and SpO2 were acquired from the alarm logs themselves. For BR since there are no threshold-based alarms, thresholds of 25 and 30 were used for red and yellow alarms respectively, in discussion with clinicians.

^d^ The choice of 50s was based on visual observations of data, as exemplified by Fig. 2 as well.

^e^ HRV features were calculated every 10s using a moving average window of the preceding 30s.

^f^ NN_Occ, in effect, is the mean value of NN-intervals in the 30s leading up to the yellow alarm. The same holds for other HRV feature based on the occurring moment.

^g^ The area under the curve is calculated using the 10^th^ percentile value of NN-intervals acting as a baseline. The same holds for the other HRV feature based on AUC.

**FIGURE 1.**
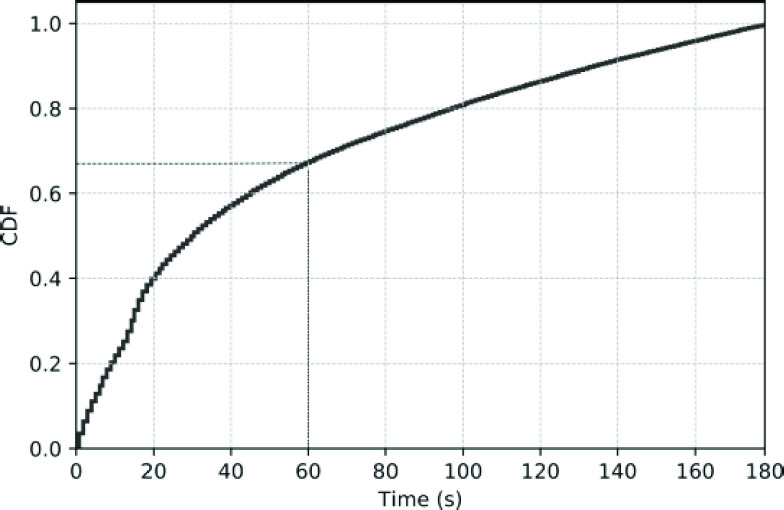
The cumulative density function (CDF) of the time to transition from all yellow to red alarms, censored at 3 minutes. Approximately, 65% of red alarms occur within 60s of a yellow alarm.

**FIGURE 2.**
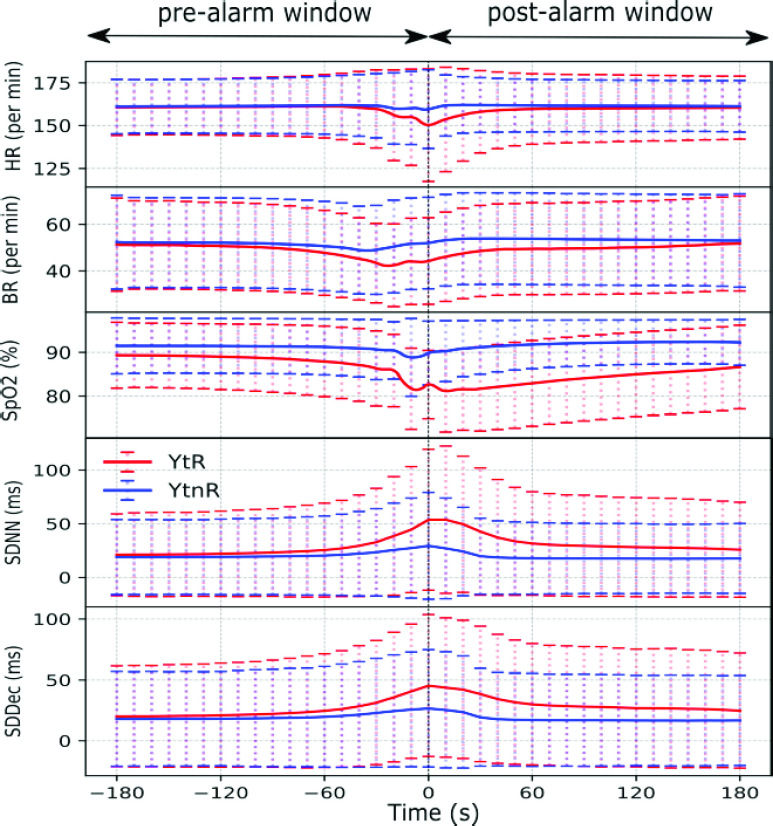
The mean and standard deviation of the HR, BR, SpO2, SDNN, and the SDDec at 10s intervals in the pre- and post-alarm windows of 3 minutes for the categories of *YtR* and *YtnR* alarms. As can be observed, there is considerable overlap between these 2 categories of alarms with differences beginning to emerge only in the 60s preceding the occurrence of the yellow alarm (represent by the black line time = 0s).

### E. Feature Generation

Based on literature and visual inspection of the time-series mentioned above (section D), eight feature-families, constituting a total of 63 features, were used for alarm prediction. These were based on, (i) *infant metadata* (3 features); (ii) *the category of yellow alarm generated* (1 feature); (iii) *the number of alarms in the pre-alarm window*(2 features); (iv) *the HR* (13 features); (v) *the BR* (13 features); (vi) *the SpO2* (13 features); (vii) *correlation between the HR, the BR and the SpO2* (6 features); and (viii) *the HRV* (12 features). A detailed account of these features is provided in Table 3. Further, we characterized the predictive potential of features from each category standalone as well as in combination with one another to identify the most important physiological data for alarm prediction.

### F. Feature Selection

All alarm data, irrespective of which infant it originated from, were randomly split into training (80%) and test (20%) sets, stratified by the proportion of *YtR* and *YtnR* alarms in the original dataset. For feature selection and parameter optimization, five-fold cross-validation (CV) within the *training set* was used with the decision tree (DT) classifier as the model of choice. The *gini* impurity index was used for splitting the tree with the maximum depth of the tree restricted to 6 levels [22]. For model derivation, for all but the first 3 feature-families (section E; i, ii, iii), a feature selection process was used to reduce the dimensionality of feature matrix by determining the smallest set of features with the most prognostic potential. For each feature-family, features were fed to the decision tree classifier in all possible combinations of 1 feature, 2 features up to n features at a time until the best performing n+1 feature combination had an area under the receiver operating characteristic (AUROC) of less than 0.001 more than the best performing n feature combination. This approach can be considered as an exhaustive feature selection process with a specific stopping criterion (AUROC increase < 0.001). For instance, for the 13 HR based features, if the best performing combination of 6 features had a mean AUROC (from CV) that was higher than the best performing combination of 5 features by 0.001 or less, the 5 best-performing features would be inducted into the combined feature-pool. This procedure of exhaustive feature selection at the feature-family level, was carried out for the aforementioned 5 feature-families (section E; iv, v, vi, vii, viii) and combined with all features from the first 3 feature-families – *infant metadata, the category of yellow alarm generated and the number of alarms in the pre-alarm window* – to constitute the final feature-pool for all 5 classifiers.

### G. Classification

After feature selection on the training set, the performance of the classifiers, per feature-family as well as for the selected feature-pool, was quantified for both the training and test sets of data using the performance metrics of AUROC and sensitivity, with the specificity fixed at 0.98. Note that for the training set, the performance was quantified as the mean value across the various folds of CV. As motivated in the introduction section, the specificity was deliberately fixed to a high value to reduce false positives. For the feature families of infant metadata, the category of yellow alarm generated and the number of yellow and red alarms in the pre-alarm window, all features were used in the calculations of the performance metrics. The motivation behind quantifying the performance of the classifiers per feature-family, before analyzing the combined feature-pool, was to generate insights into which feature-families, standalone, held the most discriminatory potential.

For the best-performing classifier, as measured by the AUROC in the test set, the corresponding decision tree up to a depth of 3 layers is presented. Next, for all 5 classifiers, an eXtreme gradient boosting (XGB) algorithm (implemented via the XGBoost library) was employed to boost the prediction performance [23]. Gradient boosting is an ensemble method that incrementally creates new trees to predict the residual errors in predictions up to the previous level, and then combines all trees to make the final improved prediction. It is called gradient boosting since it uses a gradient descent algorithm to minimize the cost function upon adding new trees to the ensemble. The first tree in this ensemble is equivalent to the unboosted decision tree. Consequent trees are tuned to predict the error in the classification of the ensemble up to that point. The prediction performance of all boosted trees is also reported. With a view towards enhancing feature interpretability, the feature importance of the top 10 features of the best-performing boosted tree (as measured by the AUROC in the test set) was calculated by parametrizing the relative contribution of each feature to the ensemble (*Gain*). The *Gain* was obtained by combining every feature’s contribution in each tree of the ensemble with a larger value reflecting higher importance for generating a prediction.

## III. Results

Overall, the database used in this study comprised of approximately 348,000 alarms (278,000 yellow alarms +70,000 red alarms), acquired from 55 infants over 34,000 patient monitoring hours. Desaturation, bradycardia and apnea alarms constituted 73%, 22% and 1% of all red alarms while the remainder were based on blood pressure (BP), tachycardia and heart fibrillation. About yellow alarms, SpO2-low, SpO2-high, HR-low, and HR-high, constituted 52%, 32%, 8% and 6% of the yellow alarms while the remainder were based on the blood pressure. For all combinations of pre- and post-alarm windows, approximately 70% of *YtR* and *YtnR* alarms satisfied the selection criterion in the pre-alarm window and were thus considered valid and included in the analysis (Table 2). Further, based on the cumulative density function of times to transition from *YtR* alarms (Fig. 1), when red alarms occur, 65% of them occur within 60s of a preceding yellow alarm.

The feature values – HR, BR, SpO2, SDNN, and SDDec – in the 3-minute windows before and after the occurrence of a yellow alarm are shown in Fig. 2 for the alarm categories of *YtR* and *YtnR*. As can be observed, there is considerable overlap in the vital signs and HRV-features between these 2 alarm-categories with differences beginning to emerge only in the 60s before the occurrence of the yellow alarm (black line), illustrating that accurate classification is challenging. From Fig. 1 and 2, we can also affirm that the choices for the pre- and post-alarm windows of up to 3 minutes, while empirical, were clinically appropriate.

Table 4 shows the performance of the decision tree classifiers for each of the 8 feature-categories as well as the combined feature-pool for all combinations of pre- and post-alarm windows, for both the training and test set of data. Overall, classification performance, as measured by AUROC and sensitivity, improved upon shortening the post-alarm window whereas changing the pre-alarm window had a limited effect on performance.

**TABLE 4:** The AUROC (Sensitivity) of the Classifier on Training and Test Sets of Data for Different Pre- and Post-Alarm Windows, for Individual Feature-Families and the Combined Feature-Pool With the Specificity Fixed at 0.98. Fot the Training Set, the Performace Results are the Mean Cross-Validated Estimate

Pre-, post-alarm window (min)	Metadata	Correlation	Alarm counts	BR	HR	HRV	Alarm category	SpO2	Combination	Total no. of combined features
Training set										
3,3	0.60 (0.05)	0.57 (0.05)	0.65 (0.07)	0.65 (0.07)	0.66 (0.09)	0.68 (0.09)	0.70 (0.06)	0.78 (0.17)	0.80 (0.21)	23
3,2	0.59 (0.04)	0.58 (0.06)	0.66 (0.07)	0.67 (0.08)	0.69 (0.12)	0.69 (0.09)	0.72 (0.07)	0.79 (0.19)	0.83 (0.22)	22
3,1	0.59 (0.04)	0.61 (0.07)	0.67 (0.07)	0.70 (0.09)	0.72 (0.14)	0.73 (0.11)	0.74 (0.08)	0.82 (0.22)	0.86 (0.29)	23
2,1	0.59 (0.04)	0.60 (0.07)	0.67 (0.07)	0.70 (0.09)	0.72 (0.13)	0.73 (0.11)	0.74 (0.08)	0.82 (0.22)	0.87 (0.29)	21
1,1	0.59 (0.04)	0.60 (0.07)	0.67 (0.07)	0.71 (0.09)	0.73 (0.13)	0.73 (0.12)	0.74 (0.08)	0.82 (0.22)	0.86 (0.29)	23
Test set										
3,3	0.60 (0.05)	0.57 (0.05)	0.65 (0.07)	0.64 (0.06)	0.66 (0.09)	0.68 (0.09)	0.70 (0.06)	0.78 (0.17)	0.80 (0.21)	23
3,2	0.59 (0.04)	0.58 (0.06)	0.66 (0.08)	0.66 (0.07)	0.68 (0.11)	0.69 (0.09)	0.72 (0.07)	0.79 (0.19)	0.83 (0.22)	22
3,1	0.59 (0.04)	0.61 (0.07)	0.67 (0.07)	0.69 (0.08)	0.72 (0.13)	0.73 (0.10)	0.74 (0.08)	0.82 (0.22)	0.86 (0.29)	23
2,1	0.59 (0.04)	0.60 (0.07)	0.67 (0.07)	0.70 (0.09)	0.72 (0.13)	0.73 (0.11)	0.74 (0.08)	0.82 (0.22)	0.87 (0.29)	21
1,1	0.59 (0.04)	0.60 (0.07)	0.67 (0.07)	0.71 (0.09)	0.73 (0.13)	0.73 (0.12)	0.74 (0.08)	0.82 (0.22)	0.86 (0.29)	23

**FIGURE 3.**
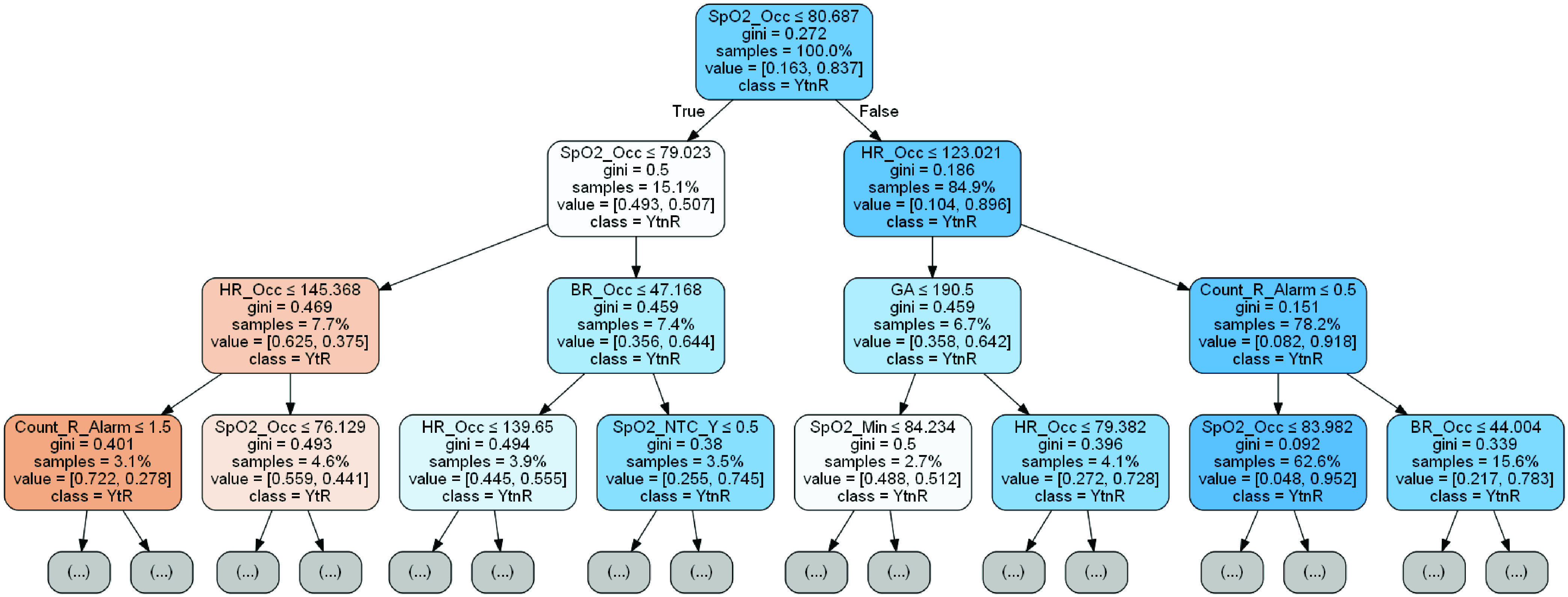
The top 3 layers of the decision tree that was trained on a pre- and the post-alarm window of 2 and 1 min respectively. The classification performance on the test set resulted in an AUROC of 0.87 and a sensitivity of 0.29 for a specificity fixed at 0.98. Features based on the SpO2 (root), HR (layer 1), BR (layer 2), infant metadata (layer 2), number of alarms in the pre-alarm window (layer 3) are shown while those based on yellow alarm category (layer 4) and HRV (layer 4) are further along the tree (not shown). The left and right arrows (branches) from each node represent the samples that meet the true and false condition tested in the node. The *gini* index represents the purity of the underlying class distribution – a low value can be interpreted as the probability of one class being higher than the other while a value close to 0.5 means both classes are equiprobable. The percentage of the original samples that are tested in each node are represented by *samples* whereas *value*[*YtR*, *YtnR*] represents the proportion of samples within the node that in fact belonged to the *class* (*YtR* or *YtnR*).

In the test set, the best-performing feature-family was based on SpO2 while a combined feature-pool of 21 features utilizing a pre-alarm window of 2 min could predict alarms in a post-alarm window of 1 min with an AUROC of 0.87 and a sensitivity of 0.29 for a specificity fixed at 0.98. The top 3 layers of the corresponding decision tree are shown in Fig. 3.

**FIGURE 4.**
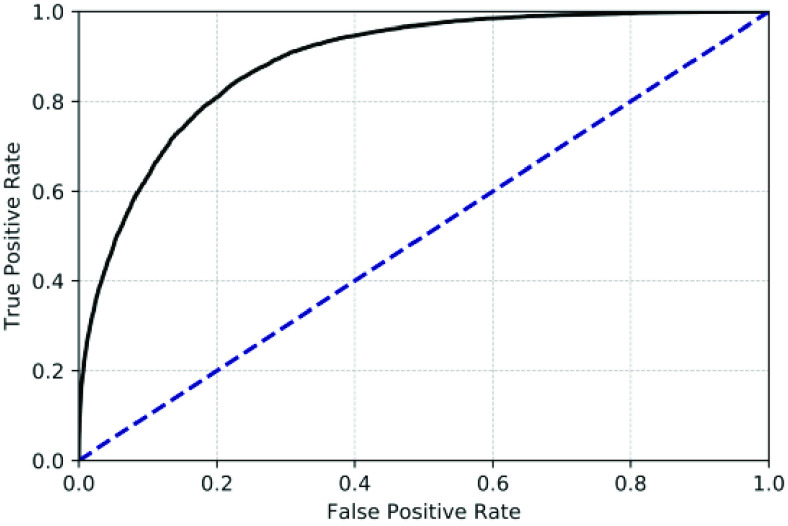
The receiver operating characteristics of the boosted decision tree for a pre- and post-alarm window of 2 and 1 min respectively. In the test set, the AUROC was 0.89 while the sensitivity was 0.33 for a specificity of 0.98.

For all 5 classifiers, the results corresponding to the performance of the boosted trees, utilizing the combined feature-pool, are quantified in Table 5. The best-performing of these classifiers had a pre-alarm window of 2 min, and a post-alarm window of 1 min. The corresponding AUROC was 0.89, and the sensitivity was 0.33 while the specificity was fixed at 0.98. The receiver operating characteristic curve of this classifier is shown in Fig. 4. For this model of the boosted tree, based on a pre- and post-alarm window of 2 and 1 min respectively, with the apriori distribution of yellow (278,000) and red (70,000) alarms, there were 232,823 cases of *YtnR* and 45,628 cases of *YtR* (= 56,415 alarms, since more than 1 red alarm in a case of *YtR* was possible). Based on the classifier performance, the ratio of true positive (56,$415\times 0.33=18$,616) to false positive (232,$823\times 0.02=4$,656) red alarms is 4:1. This implies that at the time of occurrence of the yellow alarm, for 4 red alarms correctly predicted by the classifier, 1 red alarm would be predicted incorrectly. The median (interquartile range) duration between the yellow alarm and the red alarms in the post-alarm window, i.e., the extra window of opportunity gained by early prediction was 18.4 (9.2-35.8; median and interquartile range) s.

**TABLE 5:** Performance of the Boosted Trees on the Test Set of Data for Different Pre- and Post-Alarm Windows

Pre-alarm window (min)	Post-alarm window (min)	AUROC (sensitivity)	No. of trees in the ensemble
3	3	0.85 (0.26)	184
3	2	0.86 (0.29)	184
3	1	0.89 (0.33)	136
2	1	0.89 (0.33)	108
1	1	0.89 (0.32)	104

With regard to feature importance in the best-performing boosted decision tree, all 6 yellow alarm categories, in addition to the features corresponding to values of SpO2, HR and BR at the occurring moment and the number of red alarms that occurred in the pre-alarm window constituted the 10 most important features for the boosted tree. This suggests that the decision trees in the ensemble that model the error in prediction are keying in on the differences in the feature space preceding different yellow alarm categories. This was confirmed by visualizing the 2nd, 3rd and the 4th trees of the ensemble wherein the yellow alarm category featured prominently in the top layers of the corresponding trees (figures not shown).

## IV. Discussion

This study has 5 findings of practical importance. First, when red alarms occur, they do so within a short interval of time after the occurrence of a yellow alarm (Fig. 1). Moreover, there are no marked differences in baseline values of vital signs or in the HRV preceding the occurrence of yellow alarms that lead to red alarms versus those that do not. On average, differences begin to emerge only around 60s before the occurrence of the yellow alarm (Fig. 2).

Second, based on the decision tree as a classifier, all feature-families held at least some prognostic potential to discriminate between yellow alarms that lead to a red alarm(s) versus those that don’t. Standalone, the best feature-family is based on the SpO2 (Table 4), which is reasonable since the most prevalent red alarm is desaturation (73%). The combined feature-pool performs better than individual feature-families, and the performance improves upon limiting predictions to shorter post-alarm windows, i.e., from 3 min to 1 min. This finding matches expectations since predictions further into the future are more challenging. Shortening the pre-alarm window had only a limited effect on performance since the most discriminatory features were based on data at the moment the yellow alarm occurred (Fig. 3). Notably, across feature-families as well as the combined feature-pool, the AUROC was comparable in the training and test sets (Table 4) indicating that there was little evidence of overfitting.

Third, features from 7 of the 8 feature-families, including features based on trend information, contributed to the best-performing decision tree (Fig. 3) including those from SpO2, HR, BR, infant metadata, number of alarms in the pre-alarm window, yellow alarm category and HRV. These findings showcase the relevance of employing a multiparametric approach that includes trend information for the predictive monitoring of critical alarms.

Fourth, by using boosted decision trees, the performance of alarm classification improved to an AUROC of 0.89 with a sensitivity of 0.33 while the specificity was affixed at 0.98. This improvement in performance, compared to the single best-performing decision tree can be attributed to the yellow alarm category scoring high in feature importance for the boosted classifier (ensemble of decision trees) and that the feature space splits differently depending on the category of yellow alarm that occurs. This finding can be easily reconciled – for instance, the physiological processes leading up to a SpO2-low and a SpO2-high alarm are potentially different, and this may be reflected in the physiological trends preceding the corresponding yellow alarms. Also, it is possible to exploit the fact that the apriori probability of a red alarm following a SpO2-low alarm is higher than a red alarm following a SpO2-high alarm. Herein the choice of decision trees as the classifier of choice is validated since the decision tree allows recursive partitioning of the feature space and presents a white-box and interpretable model. Moreover, decision tree-based methods, by default, can also handle missing data and do not require pre-processing of the data, enhancing the possibility of near real-time performance in clinical settings.

Considering that yellow alarms have a limited nursing response [4], they can potentially be made non-auditory (i.e., muted) or even switched off. Nevertheless, information about yellow alarms may be clinically relevant and one can opt for making them visible on the central monitor. Alternatively, the physiological instability captured by yellow alarms may be communicated through different means, for instance via composite measures of physiological stability or histograms of vital signs parameters. In principle, this is not a safety-hazard since, in the private-room NICU of this study, nurses receive only red alarms via handheld devices and may not be aware of yellow alarms unless they happen to be within the patient rooms or at the central post, which is not always the case. Therefore, our fifth finding is that if the best-performing model derived herein (boosted tree with a pre- and post-alarm window of 2 and 1 min respectively) was implemented in the NICU along with non-auditory yellow alarms, instead of 278,000 yellow and 70,000 red alarm being generated, only 74,656 red alarms would be generated of which 26% of the correctly predicted alarms (18,616) would occur 18.4s (median) earlier than in the original system. Implementing this model could reduce the total number of (auditory) alarms by nearly 80% while increasing the number of red alarms by 7%. Further, the additional window of opportunity for preemptive clinical action can have an important impact in reducing the burden of disease. Preemptively sounding red alarms implies that nurses get a longer window of opportunity to take therapeutic action that helps minimize the time infants spend with their vital signs below the thresholds for critical alarms. Algorithmically enabling this possibility is a clinically relevant development because even relatively short physiological deteriorations are associated with poor long-term outcomes, including higher mortality, increased incidence of severe intraventricular hemorrhage, bronchopulmonary dysplasia and poorer neurodevelopmental outcomes [9], [24]–[26].

The value of the predictive alarming model proposed in this work is that it serves to increase the positive predictive value of yellow alarms and may help re-sensitize nurses to alarms since the low actionability of yellow alarms in the current monitoring systems is an important factor in the desensitization of nurses [4]. However, the consequences of the small increase in the number of red alarms due to such a modeling approach will require further investigation, especially from a safety perspective. The foreseen implementation however is that such an approach would facilitate in preemptively generating a red alarm, and that for the missed alarms, the threshold-based model would still generate the usual alarm. We hypothesize that this may also lead to a reduction in clustered red alarms because of an early nursing response to the preempted red alarm [3].

Regarding the classification model of choice, based on the clinical nature of the data, we preferred to use highly interpretable models over approaches such as those based on neural networks or deep learning. Amongst interpretable models, we considered models based on (logistic) regression, the Naïve Bayes model, support vector machines (SVM) and decision trees as viable candidates. Based on clinical insights into the problem and by plotting the feature distribution(s) we could decipher that the classification problem was highly non-linear, suggesting that SVM and decision trees should be the favored models of choice owing to their capability of generating non-linear decision boundaries. The decision tree approach was chosen over SVM because of its greater interpretability, its intrinsic property of performing recursive partitioning of the feature space and because unlike SVMs it does not require parameter tuning. Computationally speaking, an advantage offered by the approach based on decision trees was that the data did not require normalization.

The intent behind the design of the model was to predict whether a red alarm would occur soon after the occurrence of a yellow alarm. However, based on the category of the yellow alarm, the features predictive of a (future) red alarm would change. This is the reason behind the improvement in performance by the use of boosted decision trees, as opposed to the use of a simple decision tree. Others have used models such as linear auto-regressive models for predicting oxygen desaturations as well as quadratic classifiers and Gaussian mixture-models for predicting apnea [27]–[29]. However, unlike the problem tackled in this paper, these works have focused on addressing acute deteriorations of a specific kind, for instance, apnea. Using a similar approach to the problem addressed in this paper would likely require training a different model for each category of yellow alarm.

A limitation of the study is that predictions for red alarms occur only at the moment that yellow alarms occur. For instance, the best-performing classifier was developed to predict red alarms that would occur within 1 min – a reasonable choice since 65% of the red alarms occur in this period (Fig. 1). Logically, for the remaining 35% of the red alarms, the yellow alarm state would persist at 60s at which point additional classifiers could be employed for predictive alarming. Another limitation of the study is that some yellow alarms owing to clinical intervention did not lead to a red alarm thus affecting the labeling of case and control data and adversely affecting the algorithm’s performance. Other limitations of the study are the absence of information on respiratory support and supplemental oxygen delivery to infants, the inclusion of which may improve model performance. Finally, incorporating other vital signs such as blood pressure may also provide additional discriminatory information.

The strengths of this observational study include employing a modest cohort of infants and a sizable number of alarms, extracted over a long period in a real-world clinical setting. Thus, the analysis by default includes, within the model’s construct, a variety of clinical and environmental profiles. Notably, unlike most machine learning applications in the biomedical context, no expert annotations were required. The absence of expert annotations implies that a model similar to the one developed in this work can be uniquely derived for any intensive care or patient monitoring settings by using the routinely monitored vital signs, ECG signal and the alarm logs of those units. In summary, the approach developed herein may be readily translated to another NICU or even other ICU settings by software updates in existing patient monitoring systems using unit-specific data. By using such an approach, the models developed would implicitly incorporate unit-specific differences such as differing thresholds of yellow alarms, differences in time delays for generating alarms and nurse-related variability in factors such as responsiveness and response times, amongst others. Further, the algorithm can be made adaptive enabling patient-specific models to be generated by allowing the algorithms to retrain based on data of individual patients.

For future work, features based on waveforms of respiration and oxygen saturation can be incorporated into the model. Additionally, estimates of infant motion, as derived from waveforms such as the ECG might provide useful information for the predictive monitoring of alarms [30]. Further, alarms from other patient monitoring devices such as ventilators can be into the modeling framework – this approach can then be used to try and reduce redundancy between patient monitor and ventilator alarms since both devices are often triggered to alarm in response to the same physiological deterioration.

## V. Conclusion

Predictive monitoring of critical cardiorespiratory alarms at subcritical thresholds of physiological variables is possible. There exists a tradeoff between the ratio of correct and false predictions of critical alarms vis-à-vis an early window of opportunity for pre-emptive clinical action. In this analysis, a quarter of all critical (red) alarms were predicted approximately 18.4s in advance at the expense of only 7% falsely predicted critical alarms providing nurses a longer response time. Inherently, this system is safe since alarms that are not predicted based on the proposed model would still be generated upon the usual breach of the threshold, as in current clinical practice.

## Appendix

See Table 6.

**TABLE 6:** Unit-Wide Default Thresholds for Alarms as Well as Delays and Averaging Times for Alarms of Different Categories

Alarm category	GA < 26 weeks	GA 26–36 weeks	GA$\ge $37 weeks	Delay (s)	Averaging times
Heart rate high (bpm)	230 (200)	230 (200)	230 (200)	0 (0)	12 RR intervals
Heart rate low (bpm)	80 (100)	80 (100)	60 (80)	0 (0)	12 RR intervals
SpO2 high (%)	n.a.(95)	n.a.(95)	n.a.(95)	n.a.(15)	10s
SpO2 low (%)	80 (85)	80 (85)	80 (92)	10 (15)	10s
Apnea time (s)	20	20	20	0	n.a.
Mean ABP high (mm Hg)	55 (50)	75 (70)	75 (70)	0	–
Mean ABP low (mm Hg)	19 (24)	25 (30)	31 (26)	0	–

Legend: Gestational age (GA), oxygen saturation (SpO2), arterial blood pressure (ABP), beats per minute (bpm). High and low refer to the high and low limits of the physiologic variable. The first value refers to the critical red alarms, and the values between brackets refer to the yellow alarms. These limits remain unchanged during patient care in our NICU.
